# The complete mitochondrial genome of a Biwa goby, *Gymnogobius isaza* (Tanaka, 1916)

**DOI:** 10.1080/23802359.2024.2368732

**Published:** 2024-06-23

**Authors:** Nao Nagao, Saki Hiraoka, Tsuyoshi Mori, Norio Shimizu, Atsushi Kurabayashi, Chiaki Kambayashi

**Affiliations:** a Faculty of Bio-Science, Nagahama Institute of Bio-Science and Technology, Shiga, Japan; b Hiroshima University Museum, Higashihiroshima, Hiroshima, Japan; c Graduate School of Science, Kyoto University, Kyoto, Japan

**Keywords:** Mitogenome, Lake Biwa, isaza, Oxudercidae

## Abstract

We determined the complete mitochondrial DNA sequence of a Biwa goby, *Gymnogobius isaza* (Tanaka, 1916) using next-generation sequencing methods. The composition of its mitogenome is the same as that observed in most other vertebrates, comprising of 13 protein-coding genes, 22 tRNA genes, two rRNA genes, and two control regions. Our molecular phylogenetic analysis confirmed the close phylogenetic relationship between *G. isaza* and *G. petschiliensis*. This mitogenome information will be useful for distribution surveys using environmental DNA and the development of conservation strategies for this species.

## Introduction

*Gymnogobius isaza* (Tanaka, 1916) of the family Oxudercidae (Kuang et al. 2018) is an endemic fish species exclusive to Lake Biwa, the largest ancient lake in Japan. Due to its rapid decline, this species has been listed as Endangered (EN) by IUCN and as Critically Endangered (CR) by Japanese Ministry of the Environment (Kanao and Hasegawa 2019; Ministry of the Environment and Government of Japan 2020). Therefore, there is urgent need to comprehend and monitor the natural habitat of this species. Environmental DNA (eDNA) has proven to be an effective tool for such distribution assessment (Bylemans et al. 2019), but the genetic information for *G. isaza* is limited to partial sequences of several mitochondrial and nuclear markers (Tabata and Watanabe 2013; Ito et al. 2021), and there are no available primer sets specifically designated for this species. In this study, we sequenced the whole mitochondrial DNA of *G. isaza* with the aim of designing specific primers for future eDNA surveys.

## Materials

**Figure 1.**
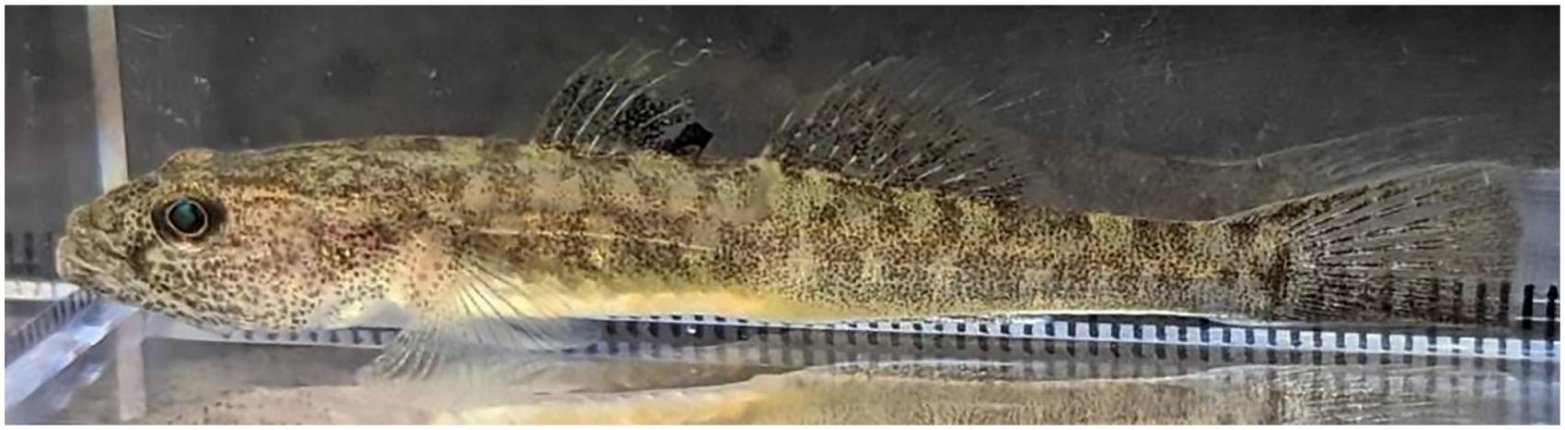
Species reference image of *Gymnogobius isaza* photographed by Tsuyoshi Mori.

The male specimen of *G. isaza* (59.4 mm in standard length) (Figure 1) was collected alive from Kaizuosaki, located north of Lake Biwa in Japan (35°26'N, 136°05'E), using nets, and deposited at the Hiroshima University Museum (contact: Norio Shimizu, museum@hiroshima-u.ac.jp) under the voucher number HUM-I-2359.

## Methods

The mtDNA  was extracted from fresh liver tissue sample isolated from the anesthetized fish specimen using the Mitochondrial DNA Isolation Kit (BioVision, CA) and sequenced on the DNBSEQ-G400 platform using a 200-bp paired-end procedure. From the resultant data, low-quality nucleotide sites (< Q30) were deleted using fastp software (Chen et al. 2018).

The whole mtDNA sequence was assembled from the remaining data using NOVOPlasty v4.3 (Dierckxsens et al. 2017) with the mtDNA of *G. urotaenia* (Genbank No. KT601093) as the reference. Gene annotation was conducted using MITOS WebServer (Bernt et al. 2013), and inaccurate gene boundaries were corrected by visual observation.

**Figure 2.**
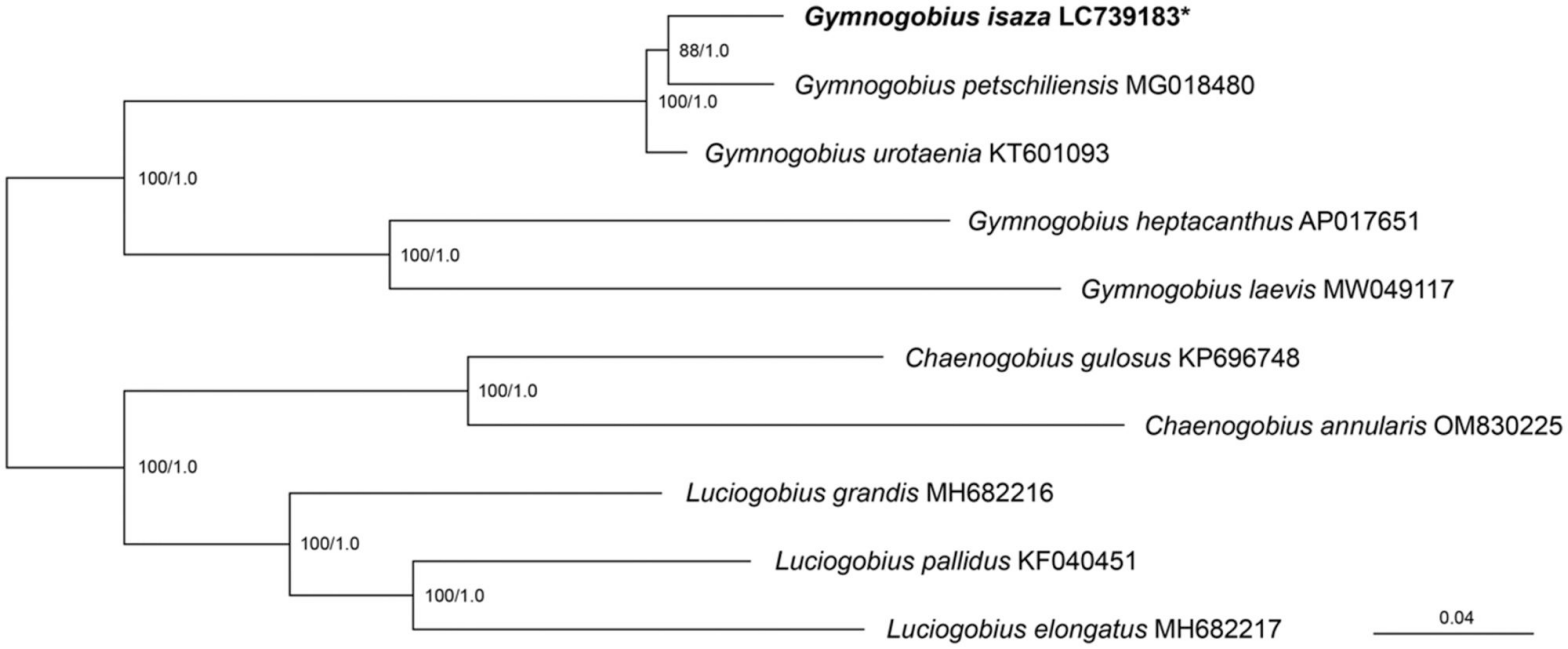
Phylogenetic tree of the oxudercid species based on mitochondrial protein-coding gene sequences. *indicates *Gymnogobius isaza* of which mtDNA was sequenced in this study. The partitioning scheme and optimal substitution models for the selected partitions were estimated by IQ-tree (partition 1 = ATP6 + ND1 + ND3 + ND4L with substitution model GTR + F + I + G4, partition 2 = ATP8 + COII + COIII with GTR + F + I + G4, partition 3 = COI + cytb with GTR + F + I + G4, partition 4 = ND2 with GTR + F + I + G4, partition 5 = ND4 with GTR + F + I + G4, partition 6 = ND5 with GTR + F + I + G4, partition 7 = ND6 with GTR + F + I + G4). The numbers at the nodes indicate maximum likelihood bootstrap values (left) and Bayesian posterior probabilities (right). Mitochondrial genome sequences are derived from the following records: *Chaenogobius annularis* OM830225 (Shang et al. 2022); *C. gulosus* KP696748 (Oh et al. 2015); *Gymnogobius heptacanthus* AP017651 (Song et al. 2016); *G. isaza* LC739183 (this study); *G. laevis* MW049117 (Peng et al. 2022); *G. petschiliensis* MG018480 (Gong et al. 2017); *G. urotaenia* KT601093 (unpublished); *Luciogobius elongatus* MH682216, *L. grandis* MH682216 (Jun et al. 2018); *L. pallidus* KF040451 (Yu et al. 2013). *Chaenogobius* and *Luciogobius* species were used as the outgroups.

Phylogenetic analyses were performed based on 13 protein-coding genes (PCGs) (11,429 bp) of 10 oxudercid species. The best-fit partitioning scheme and substitution models (see Figure 2 legend) were estimated by IQ-tree v1.6.12 (Nguyen et al. 2015). Maximum likelihood (ML) tree was reconstructed using IQ-tree, then a nonparametric bootstrapping (BS) was conducted with 1000 pseudoreplicates. Bayesian inference (BI) and posterior probabilities (PP) were obtained using MrBayes v3.2.7 (Ronquist et al. 2012) with four independent MCMC runs for two million generations. Parameter estimates and convergence of the chains were checked using Tracer v1.7.1 (Rambaut et al. 2018).

## Results

**Figure 3.**
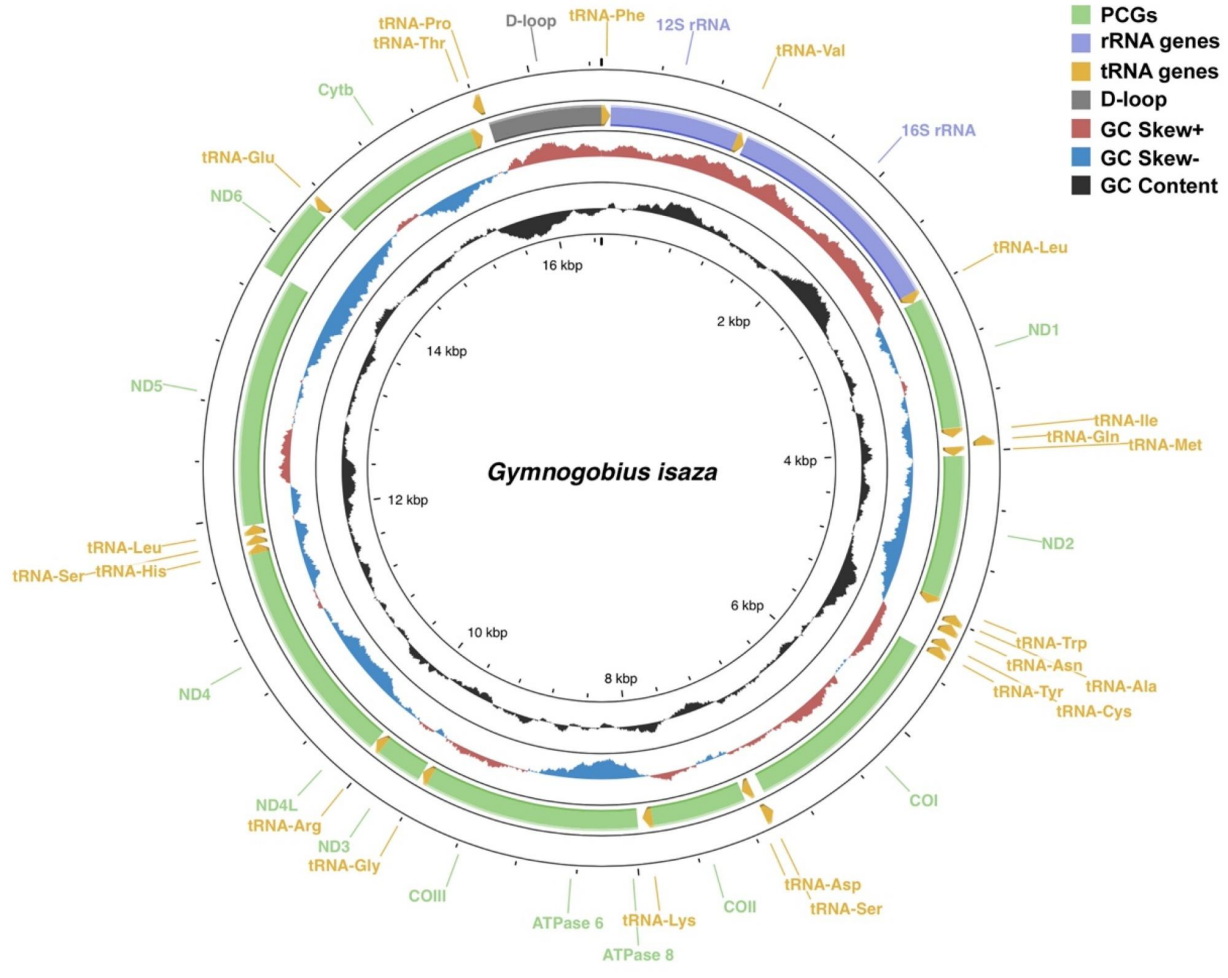
Circular sketch map of the complete mitogenome of *gymnogobius isaza*. It shows the location of protein-coding genes (PCGs), rRNA genes, and tRNA genes, as well as variations in GC nucleotide composition. Genes encoded on heavy- and light-strands are shown inside and outside the circle, respectively. Light-strand replication origin is located between the tRNA-asn and tRNA-cys genes. This map was drawn using proksee server (https://proksee.ca/).

The mtDNA of *G. isaza* is 16,477 bp in length, with an average depth of 743X (Figure S1). The mitogenome contains 13 PCGs, 22 tRNA genes, and two rRNA genes, as well as two non-coding regions, namely light-strand replication origin (O_L_) and control region (CR) (Figure 3). The gene arrangement of this species coincides with that presented in most gobioids that corresponds to the one of a typical vertebrate, in which most genes are encoded on the heavy-strand, except for the ND6 one and eight of the tRNAs (Figure 3). The nucleotide composition is 28.1, 29.3, 16.4, and 26.1% for A, T, G, and C, respectively, and the COI gene displays a non-canonical GTG start codon. In addition, five PCGs (COII–III, ND3–4, and Cytb) exhibit an incomplete stop codon for T, which can form a complete TAA stop codon through post-transcriptional polyadenylation. All tRNA genes can fold into typical cloverleaf secondary structures. The 37 bp O_L_ is located between the tRNA-Asn and tRNA-Cys genes.

Both ML and BI analyses yielded the same tree topology as shown in Figure 2. The trees indicate that *G. isaza* clustered together with *G. petschiliensis* and *G. urotaenia* (BS = 100, PP = 1.0) and formed a sister group with *G. petschiliensis* (BS = 88, PP = 1.0).

## Discussion and conclusion

The complete mitochondrial genome of *G. isaza* is 16,477 bp in length, which falls within the range of mtDNA of *Gymnogobius* species used in this study (16,422–16,529 bp), but longer than those of the closely related *G. urotaenia* and *G. petschiliensis* (Figure 2). The mtDNA of *G. isaza* contains the typical components found in vertebrates, and the composition and order of the genes are concordant with those of the congeneric species (Song et al. 2016; Gong et al. 2017; Peng et al. 2022). This represents invaluable genetic resources for future eDNA studies, enabling the distinction of *G. isaza* from its closely related species inhabiting the same water system (i.e. *G. urotaenia*) and monitoring the distribution range of the species.

Our phylogenetic analysis showed that *G. isaza* is most closely related to *G. petschiliensis*, with *G. urotaenia* as sister to this monophyletic group (i.e. *G. urotaenia* + [*G. isaza* + *G. petschiliensis*]). However, other phylogenetic hypotheses have been proposed in previous studies. For example, based on mitochondrial Cytb sequences, *G. isaza* has been suggested to be sister group of the clade comprising *G. petschiliensis* + *G. urotaenia* (Tabata and Watanabe 2013). On the other hand, in a more comprehensive multilocus study of approximately 18,000 bp combining both mitochondrial and nuclear data, *G. isaza* formed a monophyletic group with *G. urotaenia* that is sister to *G. petschiliensis* (McCraney et al. 2020). Furthermore, in the phylogenetic analyses based on 3,209 bp sequences combining mitochondrial Cytb and three nuclear markers not used in previous studies, both of the above tree topologies were yielded depending on the differences in the ML and BI analytical methods (Ito et al. 2021). The inconsistency in the phylogenetic relationships among these *Gymnogobius* species may be due to interspecific hybridization or incomplete lineage sorting as suggested for other Japanese gobiid species (Yamasaki et al. 2015). Therefore, further investigations incorporating large-scale genomic data would be required to elucidate the complicated evolutionary history of *G. isaza*.

## Supplementary Material

Supplemental Material

## Data Availability

The genome sequence data obtained in this study are openly available in GenBank (https://www.ncbi.nlm.nih.gov/) under the accession number LC739183. The associated BioProject, SRA, and Bio-Sample numbers are PRJDB14773, DRR417009, and SAMD00556828, respectively.

## References

[CIT0001] Bernt M, Donath A, Jühling F, Externbrink F, Florentz C, Fritzsch G, Pütz J, Middendorf M, Stadler PF. 2013. MITOS: improved *de novo* metazoan mitochondrial genome annotation. Mol Phylogenet Evol. 69(2):313–319. doi:10.1016/j.ympev.2012.08.023. 22982435

[CIT0002] Bylemans J, Gleeson DM, Duncan RP, Hardy CM, Furlan EM. 2019. A performance evaluation of targeted eDNA and eDNA metabarcoding analyses for freshwater fishes. Environ DNA. 1(4):402–414. doi:10.1002/edn3.41.

[CIT0003] Chen S, Zhou Y, Chen Y, Gu J. 2018. fastp: an ultra-fast all-in-one FASTQ preprocessor. Bioinformatics. 34(17):i884–i890. doi:10.1093/bioinformatics/bty560. 30423086 PMC6129281

[CIT0004] Dierckxsens N, Mardulyn P, Smits G. 2017. NOVOPlasty: *de novo* assembly of organelle genomes from whole genome data. Nucleic Acids Res. 45(4):e18. doi:10.1093/nar/gkw955. 28204566 PMC5389512

[CIT0005] Gong L, Chen W, Liu LQ, Lü ZM. 2017. The complete mitochondrial genome of *Gymnogobius petschiliensis* (Gobiiformes; Gobiidae; Gobionellinae) and its phylogenetic implications. Mitochondrial DNA B Resour. 2(2):816–818. doi:10.1080/23802359.2017.1398609. 33473994 PMC7799598

[CIT0006] Ito R, Harada S, Tabata R, Watanabe K. 2021. Molecular evolution and convergence of the rhodopsin gene in *Gymnogobius*, a goby group having diverged into coastal to freshwater habitats. J Evol Biol. 35(2):333–346. doi:10.1111/jeb.13955. 34689368

[CIT0007] Jun J, Choi SH, Kim HY. 2018. Characterization of the complete mitochondrial genomes and phylogenetic analysis of the two *Luciogobius* species (Perciformes, Gobionellinae) from Korea. Mitochondrial DNA B Resour. 3(2):1154–1155. doi:10.1080/23802359.2018.1522980. 33474449 PMC7799663

[CIT0008] Kanao S, Hasegawa K. 2019. Gymnogobius isaza. The IUCN red list of threatened species 2019. e.T110461312A110461327. doi:10.2305/IUCN.UK.2019-2.RLTS.T110461312A110461327.en.

[CIT0009] Kuang T, Tornabene L, Li J, Jiang J, Chakrabarty P, Sparks JS, Naylor GJP, Li C. 2018. Phylogenomic analysis on the exceptionally diverse fish clade Gobioidei (Actinopterygii: gobiiformes) and data-filtering based on molecular clocklikeness. Mol Phylogenet Evol. 128:192–202. doi:10.1016/j.ympev.2018.07.018. 30036699

[CIT0010] McCraney WT, Thacker CE, Alfaro ME. 2020. Supermatrix phylogeny resolves goby lineages and reveals unstable root of Gobiaria. Mol Phylogenet Evol. 151:106862. doi:10.1016/j.ympev.2020.106862. 32473335

[CIT0011] Ministry of the Environment, Government of Japan. 2020. Red list 2020 of ministry of the environment, Government of Japan. http://www.env.go.jp/press/files/jp/114457.pdf.

[CIT0012] Nguyen LT, Schmidt HA, von Haeseler A, Minh BQ. 2015. IQ-TREE: a fast and effective stochastic algorithm for estimating maximum-likelihood phylogenies. Mol Biol Evol. 32(1):268–274. doi:10.1093/molbev/msu300. 25371430 PMC4271533

[CIT0013] Oh J, Kim TW, Kim S. 2015. The complete mitochondrial genome of *Chaenogobius gulosus* (Gobiidae, Perciformes) from the South Sea, Korea. Mitochondrial DNA A DNA Mapp Seq Anal. 27(6):4207–4208. doi:10.3109/19401736.2015.1022742. 26000943

[CIT0014] Peng L, Li P, Li H, Zhao W, Wang H, Li Z, Sun L. 2022. Sequencing and analysis of the complete mitochondrial genome of *Gymnogobius laevis*. Mitochondrial DNA B Resour. 7(5):873–874. doi:10.1080/23802359.2022.2076625. 35692706 PMC9176352

[CIT0015] Rambaut A, Drummond AJ, Xie D, Baele G, Suchard MA. 2018. Posterior summarization in Bayesian phylogenetics using Tracer 1.7. Syst Biol. 67(5):901–904. doi:10.1093/sysbio/syy032. 29718447 PMC6101584

[CIT0016] Ronquist F, Teslenko M, van der Mark P, Ayres DL, Darling A, Höhna S, Larget B, Liu L, Suchard MA, Huelsenbeck JP. 2012. MRBAYES 3.2: efficient Bayesian phylogenetic inference and model choice across a large model space. Syst Biol. 61(3):539–542. doi:10.1093/sysbio/sys029. 22357727 PMC3329765

[CIT0017] Shang Y, Wang X, Liu G, Wu X, Wei Q, Sun G, Mei X, Dong Y, Sha W, Zhang H. 2022. Adaptability and evolution of Gobiidae: a genetic exploration. Animals. 12(14):1741. doi:10.3390/ani12141741.[Mismatch] 35883288 PMC9312210

[CIT0018] Song HY, Hyun YS, Yoon M, Woo J, Lim BJ, Kim HJ, An HS. 2016. The complete mitochondrial genome of *Gymnogobius heptacanthus* (Perciformes, gobiidae). Mitochondrial DNA B Resour. 1(1):833–834. doi:10.1080/23802359.2016.1247671. 33473645 PMC7800937

[CIT0019] Tabata R, Watanabe K. 2013. Hidden mitochondrial DNA divergence in the Lake Biwa endemic goby *Gymnogobius isaza*: implications for its evolutionary history. Environ Biol Fish. 96(6):701–712. doi:10.1007/s10641-012-0062-x.

[CIT0020] Yamasaki YY, Nishida M, Suzuki T, Mukai T, Watanabe K. 2015. Phylogeny, hybridization, and life history evolution of *Rhinogobius* gobies in Japan, inferred from multiple nuclear gene sequences. Mol Phylogenet Evol. 90:20–33. doi:10.1016/j.ympev.2015.04.012. 25929788

[CIT0021] Yu JN, Kim BJ, Kim S, Oh K, Lim CE. 2013. Complete mitochondrial genome of the rare hypogean gobiid, *Luciogobius pallidus*, from Korea. Mitochondrial DNA. 26(1):118–120. doi:10.3109/19401736.2013.809447. 23815332

