# Supplementary figures and images for: The complete mitochondrial genome of a Biwa goby, Gymnogobius isaza (Tanaka, 1916)
Source: Mitochondrial DNA B Resour. 2024 Jun 23;9(6):837–40. doi: 10.1080/23802359.2024.2368732 (PMC11198123; doi:10.1080/23802359.2024.2368732)

**
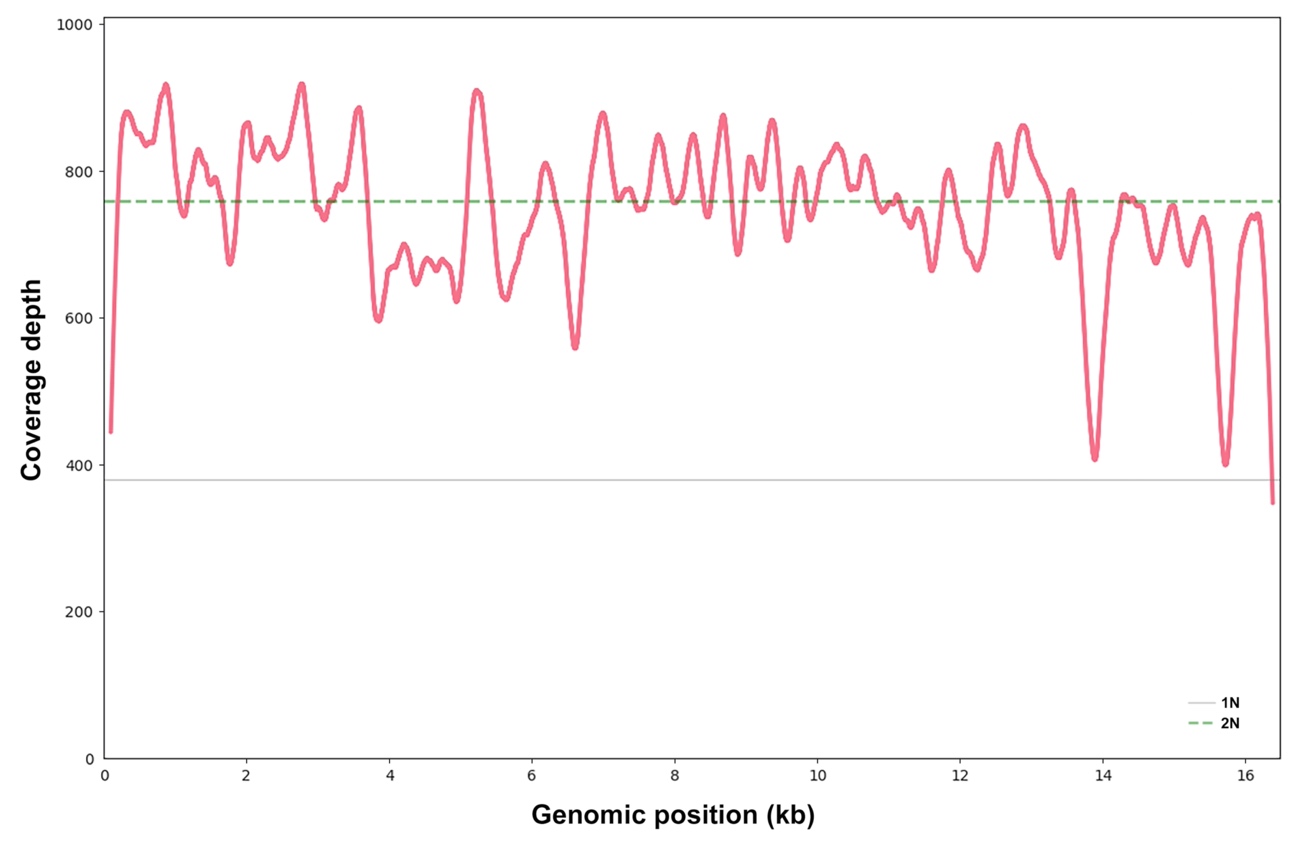
**

**Supplementary Figure 1.** The read coverage depth map of *Gymnogobius isaza*.

Supplement: Supplemental Material [file TMDN_A_2368732_SM4081.docx]
